# Strain-Dependent Assessment of Powassan Virus Transmission to *Ixodes scapularis* Ticks

**DOI:** 10.3390/v16060830

**Published:** 2024-05-23

**Authors:** Rebekah J. McMinn, Emily N. Gallichotte, Samantha Courtney, Sam R. Telford, Gregory D. Ebel

**Affiliations:** 1 Department of Microbiology, Immunology and Pathology, Colorado State University, Fort Collins, CO 80523, USA; 2 Department of Infectious Disease and Global Health, Tufts University, North Grafton, MA 01536, USA

**Keywords:** Powassan virus, ticks, transmission

## Abstract

Powassan virus (POWV) is an emerging tick-borne encephalitic virus in Lyme disease-endemic sites in North America. Due to range expansion and local intensification of blacklegged tick vector (*Ixodes scapularis*) populations in the northeastern and upper midwestern U.S., human encephalitis cases are increasingly being reported. A better understanding of the transmission cycle between POWV and ticks is required in order to better predict and understand their public health burden. Recent phylogeographic analyses of POWV have identified geographical structuring, with well-defined northeastern and midwestern clades of the lineage II subtype. The extent that geographic and genetically defined sublineages differ in their ability to infect and be transmitted by blacklegged ticks is unclear. Accordingly, we determined whether there are strain-dependent differences in the transmission of POWV to ticks at multiple life stages. Five recent, low-passage POWV isolates were used to measure aspects of vector competence, using viremic and artificial infection methods. Infection rates in experimental ticks remained consistent between all five isolates tested, resulting in a 12–20% infection rate and some differences in viral load. We confirm that these differences are likely not due to differences in host viremia. Our results demonstrate that blacklegged ticks are susceptible to, and capable of transmitting, all tested strains and suggest that the tick–virus association is stable across diverse viral genotypes.

## 1. Introduction

Tick-borne diseases are of increasing concern due to the expanding range and density of ticks and the pathogens they carry [1,2,3]. In North America, one of these pathogens is the Powassan virus (POWV; family *Flaviviridae*, genus *Flavivirus*), which can cause severe neuroinvasive disease in humans similar to its Eurasian relative, tick-borne encephalitis virus (TBEV). In the United States, POWV human cases have increased from 0.9 cases per year (1958–2007) to 18.7 cases per year (2008–2022) [4,5]. 

Lineage I POWV was originally identified in a fatal human case of encephalitis [6], and subsequently in enzootic tick vectors that infrequently bite humans. Thus, infections in humans were rare. A second lineage of the virus (lineage II) was isolated from blacklegged ticks (*Ixodes scapularis*) in the late 1990s and has since been shown to be present in the Northeast and Midwest U.S. [7,8]. Natural infection of *I. scapularis* has significant implications for human disease; they are aggressive human-biters that are regarded as the most medically important tick vector in North America, transmitting multiple other zoonotic pathogens, including *Borrelia burgdorferi*, *Babesia microti*, and *Anaplasma phagocytophilum*, the bacterial agents of Lyme disease, babesiosis, and anaplasmosis, respectively [2]. Increasing POWV seropositivity in deer [9], recent reports of high infection rates in ticks [10,11,12], and human cases reported in states with no prior history of disease [5,12,13,14] suggest dynamic transmission and epidemiology and increasing incidence of this virus in people.

Multiple phylogeographic analyses of POWV genomes suggest that the virus exists in discrete transmission foci with infrequent dispersal between enzootic sites [15,16]. Such isolation could imply adaptation to local transmission conditions such as host or vector diversity and genetic background, as well as extrinsic influences such as microclimate [17]. Indeed, geographic risk of human POWV disease appears to be heterogeneous, with Massachusetts, Wisconsin, Minnesota, Maine, and New York accounting for >90% of all cases [5]. This contrasts with the known risk for Lyme disease, with intense zoonotic transmission throughout all the New England states southward to Pennsylvania and Maryland, as well as in Wisconsin and Minnesota [18]. It may be that there is differential risk associated with local viral genotypes, as is evident for TBEV in Western Europe [19]. Accordingly, to determine whether local POWV strains may differ in their capacity to be transmitted, we measured the capacity for five isolates of POWV to infect blacklegged ticks. Specifically, we used a range of POWV strains from each lineage and from different geographic origins. and we used *I. scapularis* ticks that acquired infection either orally by feeding on mice or via immersion in virus-containing buffer. Overall, our results demonstrate the stability of the tick–virus interaction. 

## 2. Materials and Methods

***Cells and viruses***: BHK-21 (ATCC CCL-10) cells were grown in DMEM supplemented with 10% fetal bovine serum (FBS), 10 units/mL penicillin, 10 μg/mL streptomycin at 37 °C, and 5% CO_2_. Viruses (described in Table 1) were passaged on BHK cells, supernatant harvested 3–6 days post-infection, and frozen at −80 °C prior to use. 

***Plaque assays*:** Standard plaque assays were used to quantify infectious virus concentrations. Briefly, BHK cells were seeded one day prior to infection. Virus samples were rapidly thawed, serially diluted in infection media (standard growth media with 2% FBS), added to cells, and incubated at room temperature for 1 h with gentle rocking. Cells were overlaid with semi-solid 6% tragacanth media and incubated at 37 °C for five days. Cells were fixed and stained with 20% ethanol (EtOH) and 0.1% crystal violet. Plaques were counted manually.

***Mice and ticks*:** BALB/c (strain #000651) mice were obtained from Jackson Laboratories. Approval for animal protocols was obtained by the Colorado State University Institutional Animal Care and Use Committee (protocol #1257). All animal infections were conducted in Colorado State University ABSL-3 containment, according to the animal protection act. Nymphal and adult *I. scapularis* ticks were obtained from the Oklahoma State University Tick Rearing Facility. Ticks were kept in 5-dram polystyrene vials with mesh sieve tops at 24 °C with 90–95% relative humidity in glass humidity chambers with ~2 inches of saturated potassium sulfate. Larval ticks were obtained by allowing replete adult female *I. scapularis* to oviposit. Eggs were separated into ~200 egg bunches in separate vials and allowed to hatch. Ticks were washed in 70% EtOH and phosphate-buffered saline (PBS) and moved into clean vials every 3–5 weeks. 

***Sample processing, nucleic acid extraction and qRT-PCR***: Samples were homogenized in diluent (PBS supplemented with 20% FBS, 10 units/mL penicillin, 10 μg/mL streptomycin, 2.5 μg/mL amphotericin B, and 50 μg/mL gentamicin) at 24 Hz (tick samples) or 30 Hz for 2 min (mouse brain and spleen) then centrifuged at maximum speed for 10 min to pellet debris. A 50 μL sample was used for viral nucleic acid extraction using the Omega Viral DNA/RNA Kit on the KingFisher Flex instrument following manufacturer’s instructions. qRT-PCR was performed using the EXPRESS One-Step SYBR GreenER Kit (Invitrogen, Waltham, MA, USA), following the manufacturer’s instructions, with 2 μL sample volume and primers targeting the POWV NS5 gene (forward: GGCCATGACAGACACAACAGCGTTTG; reverse: GAGCGCTCTTCATCCACCAGGTTCC). Melt curves were used to confirm true POWV positives over background. A viral RNA standard covering a ~1 kB fragment of the NS5 gene was generated and used to convert cycle threshold values into viral RNA copy number, similar to as previously described [20]. Briefly, cDNA was generated from viral RNA using SuperScript IV; then, PCR was performed using Q5 polymerase with the T7 sequence incorporated into the forward primer (forward: TAATACGACTCACTATAGGGTTCAAGGTGTTGGCCC; reverse: GTTCTCCTTCCATGTACCGAAGGATCTG). RNA was generated using AmpliScribe T7 High-Yield Transcription Kit. RNA concentration was quantified then serially diluted to known copy numbers. 

***Viremic transmission***: Larval ticks (separated into vials with ~200 larvae) were infested on 15-week-old female BALB/c mice. During infestation, mice were restrained in plastic restrainers with their tails taped to prevent them from escaping. A single vial of larval *I. scapularis* was added to the nape and upper back of each mouse. The infested mouse was kept in the restrainer, loosely wrapped in paper towels, and placed in secondary containment for 30 min. Paper towels were discarded and mice were released into individual wire-bottom cages filled with ~1/2 inch of water in the bottom of the cage. The next day (day 1), mice were inoculated intraperitonially with 10^4^ or 10^2^ PFU virus (first and second experiment). Cage water was changed daily. On day 3 post-inoculation, blood was collected via retro-orbital bleed or tail snip to quantify viremia, vRNA was extracted, and qRT-PCR was performed; however, results were inconclusive and at the assay’s limit of detection. As larval ticks became replete and detached from the mice, they were collected from the water, surface-sterilized in 70% EtOH and PBS, and homogenized on day 6 or moved into 5-dram vials, as described above, until they molted into nymphs. Eight weeks post-repletion, molted nymphs were homogenized and POWV infection, and quantification was determined via qRT-PCR and plaque assay, as described above. 

***Mouse infection time course***: 15-week-old BALB/c female mice were inoculated intraperitoneally with 10^3^ PFU virus. In the first study, on days 1, 2, and 4, three mice were euthanized for each isolate. In the second study, six mice were euthanized per isolate on days 1 and 2, and three mice per isolate on day 4. In both studies, blood, brain, and spleen were collected from mice at each time point. Brain and spleen were homogenized in 10% weight/volume of diluent. Whole blood was collected in K_2_EDTA-coated tubes (BD Microtainer^®^ #365974). RNA was extracted from spleen, brain, and blood samples, and qRT-PCR was performed as described above. 

***Immersion infection method*:** Ticks were infected via the immersion method, similarly to as previously described [21]. The backs of 10-week-old BALB/c male mice were closely shaved and wiped with 70% EtOH. Foam capsules were made, as previously described [22], with 20 mm outer diameter and 12.5 mm inner diameter. The day prior to tick infestation (day −1), two foam capsules were fixed to each mouse (one on the nape and the second immediately posterior near the middle back) with a non-toxic leather adhesive (Tear Mender). Capsule attachment was checked daily and patched with glue as needed. Nymphal *I. scapularis* ticks were dehydrated overnight at 26 °C at ~45% relative humidity. The following day (−1), nymphs were immersed in 4 mL of 10^3^ PFU/mL POWV for 1 h at 37°C, with gentle vortexing every ten minutes. Nymphs were washed twice in PBS and transferred into a ventilated tube to dry overnight. The next day (day 0), 15–20 immersed nymphs were added to each capsule. Infested nymphs were checked daily and removed from the capsule when replete by cutting open the plastic capsule top and re-sealing with plastic stickers as previously described [22]. Replete nymphs were left to molt in humidity chambers, as previously described. Two weeks after the start of molting (approximately 8 weeks post-blood-feeding), adults were homogenized as described above and screened for POWV infection via qRT-PCR and plaque assay. A subset of washed, immersed nymphs was maintained in humidity chambers for 4.5 weeks then homogenized and screened for POWV vRNA via qRT-PCR, as described above. 

## 3. Results

### 3.1. Virus Isolates Used in This Study

**Figure 1:**
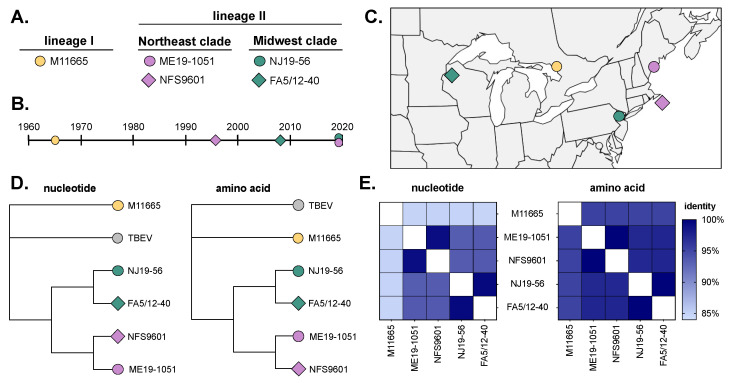
POWV isolate characteristics. (**A**) Viruses from lineage I and II (both Northeast and Midwest clades) were used in the study. Virus collection (**B**) date and (**C**) location. (**D**) Jukes–Cantor neighbor joining trees of full-genome nucleotide and amino acid sequences generated in Geneious Prime 2019.0.4. Tick-borne encephalitis virus (TBEV, NC_001672) was used as an outgroup. (**E**) Percent identity of nucleotide and amino acid sequences between isolates.

Five isolates were chosen based on geographic and temporal origin and passage history (Table 1, Figure 1). This includes a single lineage I isolate (M11665) and four lineage II isolates: two in the Northeast clade (ME19-1051 and NFS9601) and two in the Midwest clade (FA5/12-40 and NJ19-56) (Figure 1A). M11665 (lineage I; 1965) has been passaged moderately in cell culture and suckling mice; however, other isolates (ME19-1051, NJ19-56, and FA5/12-40) have been passaged in BHK cells no more than twice (Table 1). These isolates were collected over a +50-year period and from across the Northeast, the Midwest, and Canada (Figure 1B,C). Notably, though NJ19-56 was collected in New Jersey, it clusters genetically with Midwest isolates, as discussed previously [16]. The isolates in this study group phylogenetically within their lineages and clades, both at the nucleotide and amino acid level (Figure 1D). Additionally, the isolates are ~84–99% identical at the nucleotide level and over 94% identical at the amino acid level (Figure 1E).

### 3.2. Viremic Transmission to Ticks

**Figure 2:**
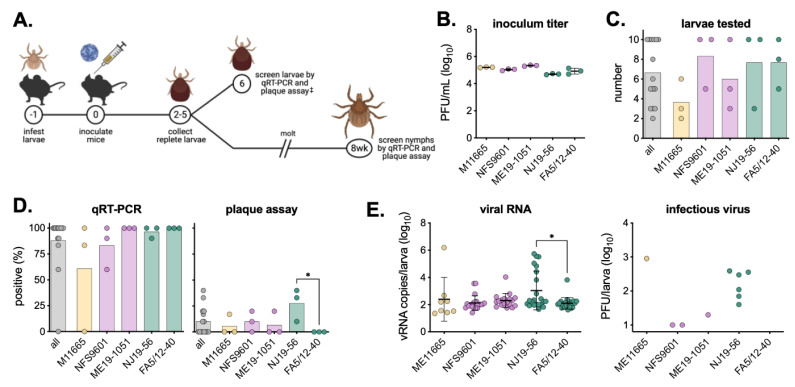
Viremic transmission of POWV to larvae. (**A**) Methodology of viremic transmission, molting, and sample collection. Numbers in circles represent study day number. ‡ Replete larvae were only collected in the first experiment. (**B**) Virus titer used to inoculate mice in the first experiment (mean ± standard deviation). (**C**) Number of replete larvae tested. (**D**) Replete larvae were tested via qRT-PCR and plaque assay to determine infection status. Symbols represent infection rates of larvae from individual mice (n = 3), with bars showing the average across mice replicates. Two-way ANOVA with Tukey’s multiple comparisons test; * *p* < 0.05. (**E**) Levels of viral RNA copies and infectious virus across different virus strains (replicate mice combined; mean ± standard deviation). Only samples with detectable viruses are plotted. Two-way ANOVA with Tukey’s multiple comparisons test; * *p* < 0.05 (insufficient data points for statistical analyses of infectious virus). Infection rates, levels of virus by individual mice, and comparisons of infection rates by individual mice compared to all larvae together are show in Supplemental Figure S1.

Viremic transmission of POWV was assessed in *I. scapularis* larvae fed on BALB/c mice infected intraperitoneally with 10^3^ PFU of each virus strain (Figure 2A,B). Approximately 200 larvae were infested a day prior to infection to overlap peak viremia with blood-feeding. Three mice were infected per isolate, and replete larvae were collected as they detached, largely on days three and four. Ten (or approximately 20%) of the replete larvae per mouse (average of 7) were homogenized and used to determine the rates and levels of virus acquisition across mice and isolates (Figure 2C). For most mice and isolates, 80–100% of larvae had detectable levels of viral RNA; however, a dramatically smaller fraction of these same larvae contained detectable infectious virus, with NJ19-56 being the highest proportion (~28%) of infectious virus detection (Figure 2D, Supplemental Figure S1A,B, Supplemental Table S1). Additionally, average levels of both vRNA and infectious virus were highest in NJ19-56-infected larvae (Figure 2E, Supplemental Figure S1C,D). Importantly, infection rates were comparable between averages across mice and averages across all replete larvae tested (Supplementary Figure S1E,F).

**Figure 3:**
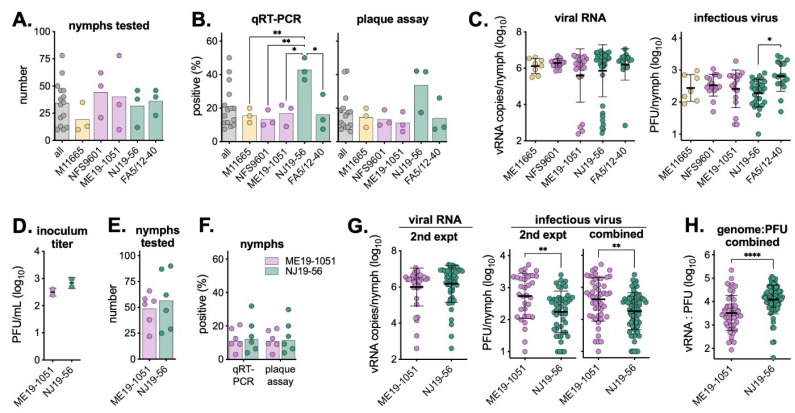
POWV infection rates and virus levels in nymphs from replete larvae. (**A**) Number of nymphs tested. (**B**) Nymphs were tested via qRT-PCR and plaque assay to determine infection status. Symbols represent infection rates of nymphs from individual mice (n = 3), with bars showing average across mice. Two-way ANOVA with Tukey’s multiple comparisons test; * *p* < 0.05, ** *p* < 0.01. (**C**) Levels of viral RNA copies and infectious virus across different virus strains (replicate mice combined; mean ± standard deviation). Only samples with detectable viruses are plotted. Two-way ANOVA with Tukey’s multiple comparisons test; * *p* < 0.05. (**D**) Virus titer used to inoculate mice in the second experiment (mean ± standard deviation). (**E**) Number of nymphs tested in the second experiment. (**F**) Nymphs were tested via qRT-PCR and plaque assay to determine infection status. Symbols represent infection rates of nymphs from individual mice (n = 6), with bars showing average across mice. The second experiment is shown individually, and data between both experiments are combined (n = 9). No comparisons between viruses in either assay were found to be significant using an unpaired *t*-test (*p* > 0.05). (**G**) Levels of viral RNA copies and infectious virus across both virus strains (replicate mice combined) in the second experiment, as well as both experiments combined (mean ± standard deviation). Only samples with detectable viruses are plotted. Two-way ANOVA with Tukey’s multiple comparisons test; ** *p* < 0.01. (**H**) Ratio of viral RNA to PFU for each nymph sample from both experiments combined (mean ± standard deviation). Unpaired *t*-test; **** *p* < 0.001. Infection rates, levels of virus by individual mice, and comparisons of infection rates by individual mice compared to all nymphs together are show in Supplemental Figure S2.

The remaining larvae were left to molt for eight weeks; then, nymphs were homogenized and screened for viral RNA and infectious virus. An average of 34 nymphs per mouse were tested (Figure 3A), with NJ19-56 having significantly higher infection rates (Figure 3B, Supplemental Table S2). Interestingly, all infected nymphs had comparable levels of viral RNA, but nymphs infected by NJ19-56 had the lowest average levels of infectious virus (Figure 3C). To better understand differences in infection rates and virus levels between lineage II Northeast and Midwest isolates, the experiment was repeated using ME19-1051 and NJ19-56. Six additional mice were infected with each isolate at a lower inoculum (Figure 3D), all replete larvae were allowed to molt into nymphs, and an average of 53 nymphs per mouse were tested for POWV infection (Figure 3E). vRNA and infectious virus infection rates were similar between both isolates (~11%), and all infected nymphs had comparable levels of vRNA (Figure 3F,G). Interestingly, in the second experiment, and when combining both experiments together, NJ19-56-infected nymphs had significantly lower levels of infectious virus (Figure 3G). Due to the similar levels of vRNA and lower levels of infectious virus, NJ19-56 has a significantly higher genome to PFU ratio than ME19-1051 (Figure 3H). Nymph infection rates and virus levels by individual mice are shown in Supplemental Figure S2.

### 3.3. Viremia in BALB/c Mice

**Figure 4:**
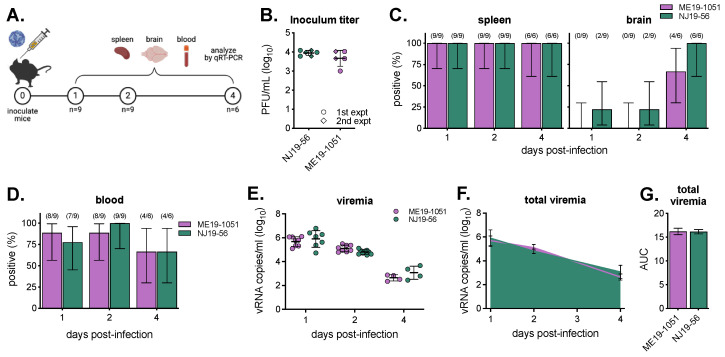
Comparison of POWV infection and viremia in mice. (**A**) Methodology of mouse infections and sample collections. Numbers in circles represent study day number. Mice groups per virus—first experiment, n = 3 on days 1, 2, and 4; 2nd experiment, n = 6 on days 1 and 2 and n = 3 on day 4. (**B**) Virus titer used to inoculate mice in both sets of experiments (mean ± standard deviation). Mouse (**C**) tissue (spleen and brain) and (**D**) blood infection rates determined via qRT-PCR (mean ± Wilson/Brown 95% confidence intervals). Number of positive samples and total number of samples tested shown above bars. No comparisons between viruses for any time point were significant using Fisher’s exact test (all *p*-values can be found in Supplemental Table S3). (**E**) Levels of viral RNA copies across both virus strains (mean ± standard deviation). Only samples with detectable viruses are plotted. Not significant via two-way ANOVA with Šidák’s multiple comparisons test (*p* > 0.05). (**F**) Area under the curve of average ME19-1051 and NJ19-56 viremia (mean ± standard deviation). (**G**) No significant differences in total viremia (area under the curve; mean ± standard error of mean) via unpaired *t*-test (*p* > 0.05).

Because we saw differences between isolates in larval infection rates and virus levels, we sought to assess potential differences in the ability of the viruses to establish viremia in the vertebrate host. In two experiments, female BALB/c mice were infected with 10^3^ PFU, and on days 1, 2, and 4 post-infection, spleen, brain, and blood were collected and tested for infection via qRT-PCR (Figure 4A,B). Across both isolates, 100% of spleens were infected by day 1; however, NJ19-56 infected the brain sooner and at higher rates than ME19-1051 (Figure 4C, Supplemental Table S3). There were significantly higher levels of NJ19-56 in the spleen on 1 day post-infection, but levels at later time points and in the brain were comparable between isolates (Supplemental Figure S3). Importantly, the percentages of mice with detectable vRNA in their blood were comparable across all time points (Figure 4C, Supplemental Table S3). Levels of viremia (as measured by viral RNA) between the two isolates were similar on all sampled days post-infection; however, its possible levels of infectious virus (which were not assayed) differed between isolates (Figure 4E). Because ticks feed on viremic mice over the course of many days, we compared the total viremia by calculating the area under the curve and saw similar levels of total virus that ticks would be exposed to (Figure 4F,G). 

### 3.4. Artificial Infection of Ticks

**Figure 5:**
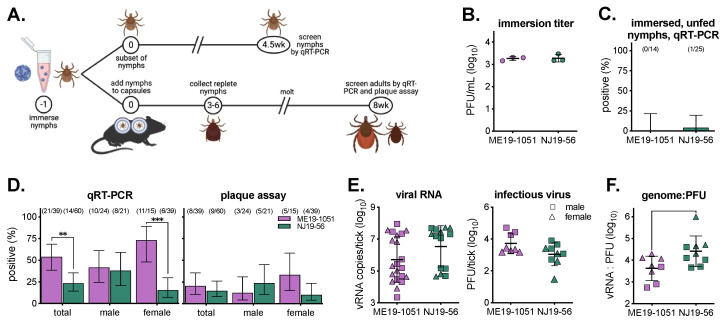
POWV infection rates and levels in artificially infected ticks. (**A**) Methodology of artificial infection, molting, and sample collection. Numbers in circles represent study day number. (**B**) Virus titer used to artificially infect ticks (mean ± standard deviation). (**C**) A subset of immersed, unfed nymphs was collected 4.5 weeks post-infection and tested for viral RNA via qRT-PCR (mean ± Wilson/Brown 95% confidence intervals). Number of positive samples and total number of samples tested are shown above bars. (**D**) Male and female adult ticks, 8 weeks post-infection, were tested for viral RNA and infectious virus via qRT-PCR and plaque assay (mean ± Wilson/Brown 95% confidence intervals). Number of positive samples and total number of ticks tested are shown above bars. Comparison of infection rates between viruses within assays were analyzed using Fisher’s exact test (** *p* < 0.005, *** *p* < 0.0001; all *p*-values in Supplemental Table S4). (**E**) Levels of viral RNA copies across both virus strains (mean ± standard deviation). Only samples with detectable viruses are plotted. No significant difference between viruses in either assay by an unpaired *t*-test (*p* > 0.05). (**F**) Ratio of viral RNA to PFU for each adult tick (mean ± standard deviation). Unpaired *t*-test; * *p* < 0.05.

To determine differences in the ability of isolates to infect ticks independent of host viremia, nymphal ticks were artificially infected. *I. scapularis* nymphs were immersed in 10^3^ PFU/mL of virus, fed on naïve mice, and left to molt for eight weeks before processing (Figure 5A,B). A subset of immersed, unfed nymphs were screened for vRNA via qRT-PCR 4.5 weeks post-immersion. Between both groups, only one tick out of 39 (2.6%) tested positive for vRNA, suggesting that higher titers in the immersion media are required to detect viruses in ticks at this timepoint (Figure 5C). After 8 weeks, molted adult ticks were homogenized and screened for vRNA and infectious virus via qRT-PCR and plaque assay. We saw significantly higher overall infection rates with ME19-1051 compared to NJ19-56 when they were analyzed via qRT-PCR, driven by differences in female infection rates (Figure 4D). However, there were no significant differences in infection rates between isolates when we measured the infectious viruses (Figure 4D, Supplemental Table S4). Between both isolates, infected adult ticks had comparable levels of viral RNA; however, ME19-1051 had higher average levels of infectious virus per tick (Figure 4E). Additionally, the genome-to-PFU ratio was significantly higher in NJ19-56-infected adults ticks compared to ME19-1051 (Figure 5F). 

## 4. Discussion

Experimental studies of POWV in ticks have been performed in *Dermacentor variabilis* [23,24], *Haemaphysalis longicornis* [25], *Amblyomma americanum* [24], and *I. scapularis* [24,26]. Notably, these studies have relied on the use of highly passaged historical virus strains. Importantly, it has been shown that *I. ricinus* ticks have different levels of susceptibility to genetically distinct TBEV strains, demonstrating a potential impact of virus genotype on tick infection [27]. With the emergence of POWV in North America, the use of low-passage, contemporary, genetically, and geographically diverse isolates is essential for an accurate representation of tick transmission. In this study, we use multiple methods to infect *I. scapularis* ticks with POWV and find efficient infection across isolates, regardless of genotype.

We sought to determine whether POWV strains differed in their ability to infect and replicate in multiple tick life-stages. Using five low-to-moderately passaged, genetically diverse POWV isolates of different geographic and temporal origins, we compared the rate of viremic transmission and infection success in a single *I. scapularis* reference colony, thus holding the tick genetic background constant. Comparable infection rates were observed between all isolates regardless of infection method, suggesting that viral mechanisms of survival and transmission within ticks may be genotype-independent. 

Ticks at multiple life stages were infected via two methods: viremic transmission (feeding on an infected mouse) and artificial infection (via immersion). During viremic transmission, the virus establishes infection in the blood of the host animal and is ingested into the midgut of the engorging tick. Artificial infection removes the variable factor of host viremia; however, blood-feeding is still necessary to induce physiological changes in the tick that promotes virus infection [28,29]. This effect was observed in our data as we were only able to detect POWV in 2.6% of unfed nymphs compared to 17.8% of blood-fed molted nymphs. Therefore, the combination of viremic and artificial infection allows us to assess strain-dependent differences in the ability of POWV isolates to infect ticks. Surprisingly, both methods resulted in comparable overall tick infection rates between 10 and 20%, similar to previously published data [24,26]. Despite the significant genetic and ecological variability of POWV, the overall tick IR was also similar between isolates, regardless of infection method. This is surprising given the number of barriers a virus must overcome to (1) develop sufficient viremia in the host mouse; (2) establish infection in the tick; and (3) survive the histolysis and rearrangement of tissues that occurs during molting [30,31,32]. With mosquito-transmitted viruses, a threshold viremia determines vector infection; although we did not define such a threshold, all strains exceeded a putative minimum viremia threshold for tick infection. 

Interestingly, we saw differences in virus infection rate and virus level in replete larvae immediately after feeding on a viremic mouse. While this might have been due to differences in host viremia, which we were unable to measure, it could also indicate differences in the ability of viral isolates to establish infection and begin replicating within the larval midgut, the first site of infection for many pathogens [33,34,35]. Additionally, it is known that tick age and density can impact feeding success and therefore virus acquisition [36]. Importantly, all isolates were successfully trans-stadially transmitted (TST). Though only a small number of larvae were screened immediately post-repletion, the proportion that were virus-positive was similar to the infection rates in nymphs post-molt, indicating low barriers to TST, as previously described [26,37]. Together, these results suggest that mechanisms of POWV infection in *I. scapularis* are not POWV genotype-dependent and are thus likely to be highly conserved.

Across multiple experiments, in multiple tick life-stages (nymphs and adults), we observed that the Midwest lineage II isolate NJ19-56 consistently had a higher genome-to-PFU ratio than the Northeast isolate ME19-1051. There are 60 amino acid differences between these isolates, potentially contributing to this or other phenotypes (Supplemental Figure S4). Differences in specific infectivities have been seen across diverse virus families and strains and indicate that NJ19-56 has more defective genomes, more defective/non-infectious particles, is less thermally stable, etc. [38,39]. Future experiments should characterize these aspects of virus biology to determine if viral sequence, origin, passage history, etc., contribute to differences in specific infectivity. It is important to know and quantify any differences in genome-to-infectious-virus ratios across virus strains as this could confound POWV PCR-based tick surveillance efforts and infection rates, which might not be representative of true infection, and therefore risk to humans [40,41].

We also assessed the infection rate and viral load of adult male and female ticks infected via immersion as nymphs. Previously, we reported significantly higher rates of POWV infection in field-collected females compared to males from the Northeast U.S. [16]. In this study, we again observed significantly higher rates of viral RNA detection in females compared to males; however, there were no differences in the detection of infectious viruses or levels of virus by sex. Biological factors, such as the difference in sex organs and the size and function of the midgut and salivary glands, could influence virus infection and replication [32]. Thus, additional work is needed to determine virus infection, replication, and tissue tropism in adult ticks. Additionally, we observed a bimodal distribution of viral RNA in infected adult ticks (both male and female), with infectious virus only recoverable from samples with high levels of viral RNA. In the samples with low levels of viral RNA, it is possible that we are detecting infected cells with residual viral RNA and not infectious virions. Future experiments should evaluate whether ticks with low levels of viral RNA are able to transmit viruses either to a naïve host or trans-stadially to the next tick life-stage.

It has been shown that different isolates can have different levels of viremia in vertebrate models of infection and pathogenesis [42,43,44]. While infecting ticks via a viremic host is most similar to how they become infected in nature, these differences in viremia can confound potential differences in tick susceptibility to different virus strains and isolates because ticks acquire varying amounts of infectious virus during feeding. Therefore, ex vivo midgut and salivary gland cultures, backless tick models, and artificial membrane feeding could be utilized to better understand how genetic differences between POWV strains impact various aspect of infection and replication within tick tissues and cells [45,46,47,48]. 

In conclusion, we report consistent rates of infected ticks between diverse viral isolates via viremic and artificial infection. The minimal strain-dependent differences in tick transmission that we observed for POWV suggest that such variation is not a basis for differential human risk (i.e., sites where POWV is expected based on the enzootic presence of the virus and intense Lyme disease risk, but human cases are rarely reported). Variation in enzootic viral transmission may be more closely associated with variables related to the vertebrate host or aspects of the tick–virus interaction (e.g., vertical transmission efficiency) not directly addressed by our studies.

## Figures and Tables

**Table 1:** POWV isolates characteristics. SM = suckling mice; V = Vero cells; B = BHK cells; P = unknown passage.

Isolate ID	Lineage	Location	Year	Source	Passage History	GenBank Accession
M11665	I	Ontario	1965	*I. cookei*	P1SM1V1B1	OP823404
NFS9601	II	Nantucket, MA	1996	*I. scapularis*	SM1B2	HM440559
ME19-1051	II	Cape Elizabeth, ME	2019	*I. scapularis*	B1	OP823442
FA5/12-40	II	Spooner, WI	2008	*I. scapularis*	B2	OP823475
NJ19-56	II	Hardwick, NJ	2019	*I. scapularis*	B1	OP823460
M11665	I	Ontario	1965	*I. cookei*	P1SM1V1B1	OP823404

## Data Availability

Data are contained within the article or Supplementary Materials.

## Supplementary Materials

The following supporting information can be downloaded at https://www.mdpi.com/article/10.3390/v16060830/s1: Figure S1: Infection rates and virus levels of replete larvae by individual mice; Figure S2: Infection rates and virus levels of nymphs by individual mice; Figure S3: Mouse brain and spleen viral RNA levels; Figure S4: Amino acid differences between ME19-1051 and NJ19-56; Table S1: Fisher’s exact test of replete larvae infection rates; Table S2: Fisher’s exact test of nymph infection rates; Table S3: Fisher’s exact test of mouse tissue and blood infection rates; Table S4: Fisher’s exact test of adult tick infection rates; Table S5: Age, sex and weights of mice used in experiments.
