# Supplementary material for: Strain-Dependent Assessment of Powassan Virus Transmission to Ixodes scapularis Ticks
Source: Viruses. 2024 May 23;16(6):830. doi: 10.3390/v16060830 (PMC11209038; doi:10.3390/v16060830)
Supplement: Supplementary file 1 [file viruses-16-00830-s001.zip › viruses-3008303-supplementary.pdf]

Supplemental Figures and Tables

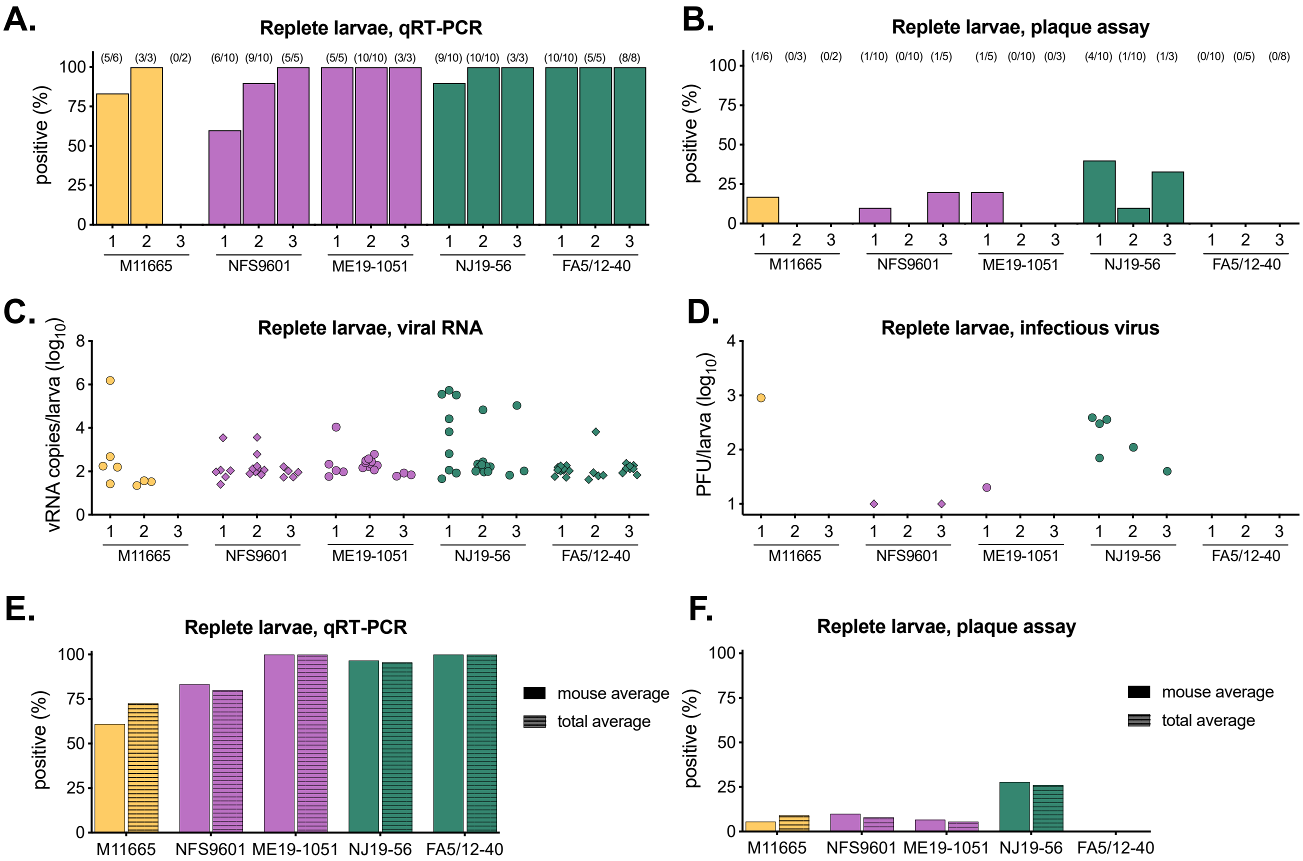

Supplemental Figure S1. Infection rates and virus levels of replete larvae by individual mice. Replete larvae from each mouse (3 per virus) were tested via **A)** qRT-PCR and **B)** plaque assay to determine infection status. Number of positive and total number of samples tested shown above bars. Levels of **C)** viral RNA copies and **D)** infectious virus across individual mice. Only samples with detectable virus are plotted. Comparison of average **E)** qRT-PCR and **D)** plaque assay infection rates across replicate mice, compared to the average of all larvae together.

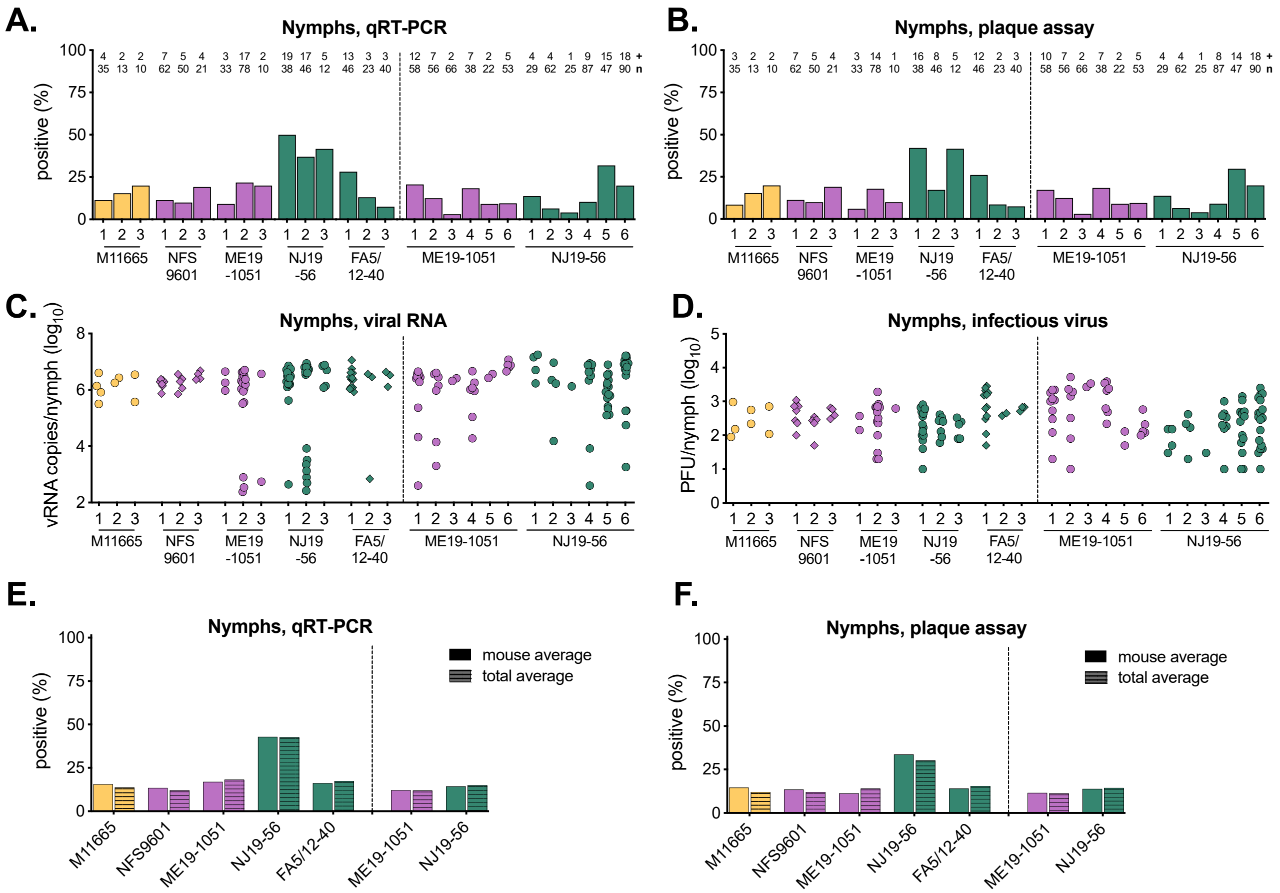

Supplemental Figure S2. Infection rates and virus levels of nymphs by individual mice. Nymphs from each mouse (1st experiment - 3 per virus, 2nd experiment – 6 per virus) were tested via **A)** qRT-PCR and **B)** plaque assay to determine infection status. Number of positive and total number of samples tested shown above bars. Levels of **C)** viral RNA copies and **D)** infectious virus across individual mice. Only samples with detectable virus are plotted. Comparison of average **E)** qRT-PCR and **D)** plaque assay infection rates across replicate mice, compared to the average of all nymphs together.

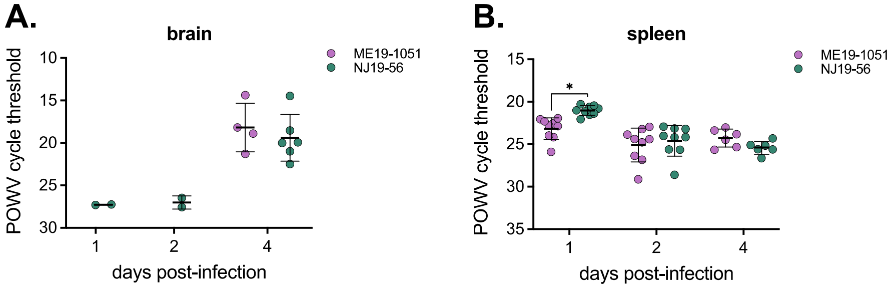

Supplemental Figure S3. Mouse brain and spleen viral RNA levels. POWV cycle threshold of infected **A)** brain and **B)** spleen tissues. Only samples with detectable vRNA are plotted. Differences between viruses at each time point were compared using multiple un-paired t-tests (*p<0.05).

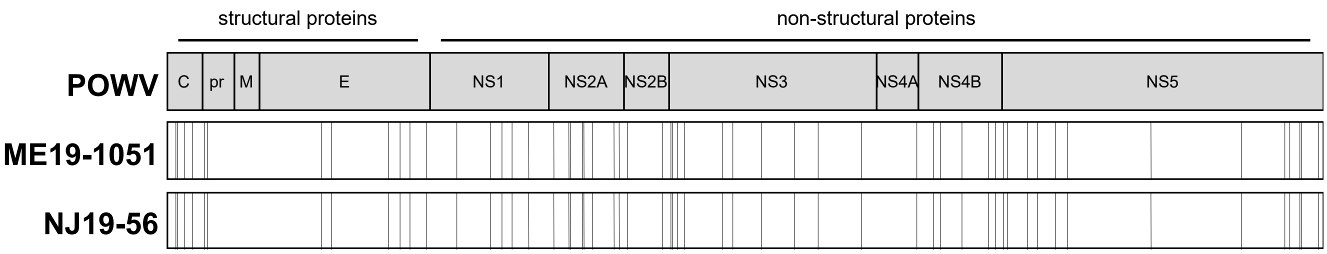

Supplemental Figure S4. Amino acid differences between ME19-1051 and NJ19-56. ME19-1051 and NJ19-56 amino acid sequences were compared. Thin lines on denote differences between ME19-1051 and NJ19-56 (60 aa changes total).

Supplemental Table S1. Fisher's exact test of replete larvae infection rates.

	M11665			NFS9601			ME19-1051			NJ19-56			FA5/12-40			Fisher's exact test	
assay	pos	neg	total	pos	neg	total	pos	neg	total	pos	neg	total	pos	neg	total	significance	p-value
qRT-PCR	8	3	11	20	5	25	18	0	18	22	1	23	23	0	23	**	0.007
plaque assay	1	10	11	2	23	25	1	17	18	6	17	23	0	23	23	*	0.0441

Supplemental Table S2. Fisher's exact test of nymph infection rates.

		M11665			NFS9601			ME19-1051			NJ19-56			FA5/12-40			Fisher's exact test	
	assay	pos	neg	total	pos	neg	total	pos	neg	total	pos	neg	total	pos	neg	total	significance	p-value
1st experiment	qRT-PCR	8	50	58	16	117	133	22	99	121	41	55	96	19	90	109	****	<0.0001
	plaque assay	7	51	58	16	117	133	18	103	121	29	67	96	17	92	109	**	0.0067
2nd experiment	qRT-PCR							35	258	293	51	289	340				ns	0.2957
	plaque assay							33	260	293	50	290	340				ns	0.2377
combined experiments	qRT-PCR	8	50	58	16	117	133	57	357	414	92	344	436	19	90	109	*	0.0287
	plaque assay	7	51	58	16	117	133	51	363	414	79	357	436	17	92	109	ns	0.1467

Supplemental Table S3. Fisher's exact test of mouse tissue and blood infection rates.

		ME19-1056			NJ19-56			Fisher's exact test	
**sample**	**day**	**pos**	**neg**	**total**	**pos**	**neg**	**total**	**significance**	**p-value**
**blood**	1	8	1	9	7	2	9	ns	>0.9999
	2	8	1	9	9	0	9	ns	>0.9999
	4	4	2	6	4	2	6	ns	>0.9999
**spleen**	1	9	0	9	9	0	9	ns	>0.9999
	2	9	0	9	9	0	9	ns	>0.9999
	4	6	0	6	6	0	6	ns	>0.9999
**brain**	1	0	9	9	2	7	9	ns	0.4706
	2	0	9	9	2	7	9	ns	0.4706
	4	4	2	6	6	0	6	ns	0.4545

Supplemental Table S4. Fisher's exact test of adult tick infection rates.

		ME19-1056			NJ19-56			Fisher's exact test	
**assay**	**group**	**pos**	**neg**	**total**	**pos**	**neg**	**total**	**significance**	**p-value**
**qRT-PCR**	total	21	18	39	14	46	60	**	0.0026
	male	10	14	24	8	13	21	ns	>0.9999
	female	11	4	15	6	33	39	***	0.0001
**plaque assay**	total	8	31	39	9	51	60	ns	0.5873
	male	3	21	24	5	16	21	ns	0.4427
	female	5	10	15	4	35	39	ns	0.0958

Supplemental Table S5. Age, sex and weights of mice used in experiments.

	number	isolate	mouse #	strain	age	sex	approx. starting weight
**Figure 2. Viremic transmission**	1	mock	1	BALB/c	15-week old	female	20-25g
	2	mock	2	BALB/c	15-week old	female	20-25g
	3	NJ19-56	1	BALB/c	15-week old	female	20-25g
	4	NJ19-56	2	BALB/c	15-week old	female	20-25g
	5	NJ19-56	3	BALB/c	15-week old	female	20-25g
	6	ME19-1051	1	BALB/c	15-week old	female	20-25g
	7	ME19-1051	2	BALB/c	15-week old	female	20-25g
	8	ME19-1051	3	BALB/c	15-week old	female	20-25g
	9	NFS9601	1	BALB/c	15-week old	female	20-25g
	10	NFS9601	2	BALB/c	15-week old	female	20-25g
	11	NFS9601	3	BALB/c	15-week old	female	20-25g
	12	M11665	1	BALB/c	15-week old	female	20-25g
	13	M11665	2	BALB/c	15-week old	female	20-25g
	14	M11665	3	BALB/c	15-week old	female	20-25g
	15	FA5/12-40	1	BALB/c	15-week old	female	20-25g
	16	FA5/12-40	2	BALB/c	15-week old	female	20-25g
	17	FA5/12-40	3	BALB/c	15-week old	female	20-25g

	number	isolate	mouse #	strain	age	sex	approx. starting weight
**Figure 3. Viremic transmission**	1	NJ19-56	1	BALB/c	15-week old	female	20-25g
	2	NJ19-56	2	BALB/c	15-week old	female	20-25g
	3	NJ19-56	3	BALB/c	15-week old	female	20-25g
	4	NJ19-56	4	BALB/c	15-week old	female	20-25g
	5	NJ19-56	5	BALB/c	15-week old	female	20-25g
	6	NJ19-56	6	BALB/c	15-week old	female	20-25g
	7	ME19-1051	1	BALB/c	15-week old	female	20-25g
	8	ME19-1051	2	BALB/c	15-week old	female	20-25g
	9	ME19-1051	3	BALB/c	15-week old	female	20-25g
	10	ME19-1051	4	BALB/c	15-week old	female	20-25g
	11	ME19-1051	5	BALB/c	15-week old	female	20-25g
	12	ME19-1051	6	BALB/c	15-week old	female	20-25g

	collection day	isolate	mouse #	strain	age	sex	starting weight
**Figure 4. Mouse infection, experiment #1**	1	NJ19-56	1	BALB/c	15-week old	female	22g
	1	NJ19-56	2	BALB/c	15-week old	female	23g
	1	NJ19-56	3	BALB/c	15-week old	female	23g
	1	ME19-1051	1	BALB/c	15-week old	female	23g
	1	ME19-1051	2	BALB/c	15-week old	female	27g
	1	ME19-1051	3	BALB/c	15-week old	female	22g
	2	NJ19-56	1	BALB/c	15-week old	female	22g
	2	NJ19-56	2	BALB/c	15-week old	female	23g
	2	NJ19-56	3	BALB/c	15-week old	female	22g
	2	ME19-1051	1	BALB/c	15-week old	female	22g
	2	ME19-1051	2	BALB/c	15-week old	female	26g
	2	ME19-1051	3	BALB/c	15-week old	female	19g
	4	NJ19-56	1	BALB/c	15-week old	female	22g
	4	NJ19-56	2	BALB/c	15-week old	female	24g
	4	NJ19-56	3	BALB/c	15-week old	female	21g
	4	ME19-1051	1	BALB/c	15-week old	female	21g
	4	ME19-1051	2	BALB/c	15-week old	female	24g
	4	ME19-1051	3	BALB/c	15-week old	female	22g
	---	mock	1	BALB/c	15-week old	female	23g
	---	mock	2	BALB/c	15-week old	female	21g
	---	mock	3	BALB/c	15-week old	female	26g

	collection day	isolate	Mouse #	strain	age	sex	starting weight
**Figure 4. Mouse infection, experiment #2**	1	NJ19-56	1	BALB/c	15-week old	female	26g
	1	NJ19-56	2	BALB/c	15-week old	female	23g
	1	NJ19-56	3	BALB/c	15-week old	female	26g
	1	NJ19-56	4	BALB/c	15-week old	female	22g
	1	NJ19-56	5	BALB/c	15-week old	female	23g
	1	NJ19-56	6	BALB/c	15-week old	female	21g
	1	ME19-1051	1	BALB/c	15-week old	female	25g
	1	ME19-1051	2	BALB/c	15-week old	female	22g
	1	ME19-1051	3	BALB/c	15-week old	female	24g
	1	ME19-1051	4	BALB/c	15-week old	female	20g
	1	ME19-1051	5	BALB/c	15-week old	female	23g
	1	ME19-1051	6	BALB/c	15-week old	female	24g
	2	NJ19-56	1	BALB/c	15-week old	female	25g
	2	NJ19-56	2	BALB/c	15-week old	female	21g
	2	NJ19-56	3	BALB/c	15-week old	female	27g
	2	NJ19-56	4	BALB/c	15-week old	female	24g
	2	NJ19-56	5	BALB/c	15-week old	female	23g
	2	NJ19-56	6	BALB/c	15-week old	female	25g
	2	ME19-1051	1	BALB/c	15-week old	female	23g
	2	ME19-1051	2	BALB/c	15-week old	female	22g
	2	ME19-1051	3	BALB/c	15-week old	female	23g
	2	ME19-1051	4	BALB/c	15-week old	female	22g
	2	ME19-1051	5	BALB/c	15-week old	female	25g
	2	ME19-1051	6	BALB/c	15-week old	female	25g
	4	NJ19-56	1	BALB/c	15-week old	female	24g
	4	NJ19-56	2	BALB/c	15-week old	female	25g
	4	NJ19-56	3	BALB/c	15-week old	female	20g
	4	ME19-1051	1	BALB/c	15-week old	female	23g
	4	ME19-1051	2	BALB/c	15-week old	female	25g
	4	ME19-1051	3	BALB/c	15-week old	female	22g
	**number**	**isolate**	**mouse #**	**strain**	**age**	**sex**	**approx. starting weight**
**Figure 5. Immersed ticks**	1	NJ19-56	1	BALB/c	10-week old	male	20-25g
	2	NJ19-56	2	BALB/c	10-week old	male	20-25g
	3	NJ19-56	3	BALB/c	10-week old	male	20-25g
	4	NJ19-56	4	BALB/c	10-week old	male	20-25g
	5	ME19-1051	1	BALB/c	10-week old	male	20-25g
	6	ME19-1051	2	BALB/c	10-week old	male	20-25g
	7	ME19-1051	3	BALB/c	10-week old	male	20-25g
	8	ME19-1051	4	BALB/c	10-week old	male	20-25g
